# PRPF6 promotes androgen receptor/androgen receptor-variant 7 actions in castration-resistant prostate cancer cells

**DOI:** 10.7150/ijbs.50810

**Published:** 2021-01-01

**Authors:** Wei Liu, Chunyu Wang, Shengli Wang, Kai Zeng, Shan Wei, Ning Sun, Ge Sun, Manlin Wang, Renlong Zou, Wensu Liu, Lin Lin, Huijuan Song, Zining Jin, Yue Zhao

**Affiliations:** 1 Department of Cell Biology, Key laboratory of Cell Biology, Ministry of Public Health, and Key laboratory of Medical Cell Biology, Ministry of Education, School of Life Sciences, China Medical University, Shenyang City, Liaoning Province110122, China.; 2 Department of Molecular Oncology, Liao Ning Tumor Hospital, Shenyang, Liaoning 110042, China.; 3 Department of Breast Surgery, the First Affiliated Hospital of China Medical University, Shenyang City 110001, Liaoning Province, China.

**Keywords:** androgen receptor, pre-mRNA processing factor 6, alternative splicing, transcriptional regulation, prostate cancer

## Abstract

Androgen receptor (AR) and its variants play vital roles in development and progression of prostate cancer. To clarify the mechanisms involved in the enhancement of their actions would be crucial for understanding the process in prostate cancer and castration-resistant prostate cancer transformation. Here, we provided the evidence to show that pre-mRNA processing factor 6 (PRPF6) acts as a key regulator for action of both AR full length (AR-FL) and AR variant 7 (AR-V7), thereby participating in the enhancement of AR-FL and AR-V7-induced transactivation in prostate cancer. In addition, PRPF6 is recruited to *cis*-regulatory elements in AR target genes and associates with JMJD1A to enhance AR-induced transactivation. PRPF6 also promotes expression of AR-FL and AR-V7. Moreover, PRPF6 depletion reduces tumor growth in prostate cancer-derived cell lines and results in significant suppression of xenograft tumors even under castration condition in mouse model. Furthermore, PRPF6 is obviously highly expressed in human prostate cancer samples. Collectively, our results suggest PRPF6 is involved in enhancement of oncogenic AR signaling, which support a previously unknown role of PRPF6 during progression of prostate cancer and castration-resistant prostate cancers.

## Introduction

Prostate cancer is one of the leading causes of male cancer-related deaths worldwide 1. The growth of prostate cancer is controlled by androgen/androgen receptor (AR) axis. Androgen depletion therapy is the primary treatment for advanced prostate cancer and initially effective for tumor suppression 2. However, a majority of patients eventually progress to a hormone resistant or castration-resistant prostate cancer (CRPC) within 2-3 years. Thus, improved therapeutic target for CRPC remains an urgent requirement.

AR acting as a hormone inducible transcription factor is a member of the nuclear receptor superfamily. Full-length AR (AR-FL) mainly consists of ligand-independent activation function (AF-1) and ligand-dependent activation function (AF-2). By alternative splicing process, C-terminal truncated variants of AR (AR-Vs) are generated from *AR* gene. AR-Vs are constitutively active and predominantly distributed in the nucleus, allowing them to drive gene transcription independent of ligand binding 3, 4. AR-FL and AR-Vs along with the downstream signals are considered as the key drivers for CRPC progression 5, 6. Accumulating evidences demonstrate that CRPC remains dependent on persistent AR signaling, which is driven by increased androgen synthesis, enhanced nuclear transport of AR, activated *AR* enhancer, *AR* amplification, overexpression of AR co-regulators, and generation of AR-Vs 5, 7-10. It has been considered that aberrant expression of AR-FL/AR-Vs co-regulators lead to prostate cancer as well as CRPC via the abnormal function of co-regulators on modulation of AR transcriptional network 11-14. AR-V7 is, to date, considered to be the putative AR variant. Importantly, AR-V7 is rarely expressed in primary prostate cancer, but the expression of AR-V7 is increased in CRPC, serving as an independent predictive factor for CRPC development and cancer specific survival 15-17. AR-V7 generation has generally been attributed to alternative splicing of *AR* pre-mRNA and/or *AR* genomic structural rearrangement 3, 4, 18, 19. Thus, to clarify the mechanisms involved AR-V7 generation as well as AR-FL/AR-V7 co-regulators would be crucial for understanding prostate cancer and the process of CRPC transformation.

In eukaryotic cells, mature messenger RNAs are formed by splicing from nascent precursor messenger RNAs, which is operated by spliceosomes 6. Spliceosomal proteins can be aberrant expressed or somatic mutated in many cancers, and play vital roles in cancer development and progression 20-22. Pre-mRNA processing factor 6 (PRPF6) is a component of spliceosome, and plays a role in the formation of spliceosome 23. The study concerning the function of PRPF6 in tumor is rarely reported. It has been demonstrated that PRPF6 drives cancer proliferation by preferential splicing of genes associated with cell growth in colorectal carcinoma 24. In addition to the function of PRPF6 on splicing, our study has previously demonstrated that PRPF6 interacts with the N-terminus of AR and enhances AR-mediated transactivation 25. However, little work has been done to understand the molecular mechanism underlying the modulation function of PRPF6 on AR action and the role of PRPF6 in prostate cancer.

Here, our results have demonstrated PRPF6 acts as a key regulator for action of both AR-FL and AR-V7, thereby participating in the enhancement of AR-FL and AR-V7-induced transactivation in prostate cancer. In addition, PRPF6 is recruited to *cis*-regulatory elements in AR target genes and associates with JMJD1A to enhance AR-induced transactivation. PRPF6 also promotes expression of AR-FL and AR-V7. Moreover, PRPF6 depletion reduces tumor growth in prostate cancer-derived cell lines and results in significant suppression of xenograft tumors even under castration condition in mouse model. Furthermore, PRPF6 is obviously highly expressed in human prostate cancer samples. Taken together, our results demonstrate that PRPF6 is involved in enhancement of oncogenic AR signaling, which provides an insight to potential therapeutic strategies for prostate cancer, especially for CRPC.

## Materials and Methods

### Plasmids

Luciferase reporter plasmids and expression plasmids of Myc-tagged PRPF6, AR-FL and AR-V7 were described previously 13, 25. For FLAG-tagged full length and truncated mutants of PRPF6, the encoding cDNAs were cloned into plasmid vector pcDNA3-FLAG.

### Antibodies and reagents

The antibodies used in this study were: anti-PRPF6 (Bethyl, A302-773A; Santa Cruz, sc-166889; Proteintech, 23929-1-AP), anti-AR (Invitrogen, MA5-13426; Proteintech, 22089-1-AP), anti-MLL1 (Bethyl, A300-37A), anti-JMJD1A (Proteintech, 12835-1-AP), anti-PSA (Proteintech, 10679-1-AP), anti-FASN (Proteintech, 10624-2-AP), anti-UBE2C (Proteintech, 66087-1-lg), anti-H3K4me3 (Sigma-Aldrich, 05-745R), anti-H3K9me2 (Sigma-Aldrich, 05-1249), anti-H3K36me3 (Millipore, ABE435), anti-FLAG (Shanghai Genomics, GNI4110-FG), anti-Myc (Shanghai Genomics, GNI4110-MC), anti-β-Actin (Proteintech 60008-1-Ig), anti-GAPDH (ABclonal, AC033). Other reagents used in this study were: normal mouse IgG (Santa Cruz, sc-2025), normal rabbit IgG (Santa Cruz, 2027). Transfections of plasmids and siRNAs were performed according to manufacturer's instructions of jetPRIME reagents (Polyplus-transfection). Charcoal/dextran-stripped serum (CSS) was prepared from fetal bovine serum (FBS, CLARK) according to the standard protocol.

### Cell culture

Human PCa cell line LNCaP, CWR22Rv1 and VCaP were obtained from Kunming cell bank of Chinese Academy of Sciences, and were identified using PCR-STR analysis. LNCaP, CWR22Rv1 and VCaP cells were cultured in Roswell Park Memorial Institute medium 1640 (RPMI-1640, Gibco) with 10% (v/v) FBS (CLARK). HEK293, cells were cultured in Dulbecco's modified Eagle medium (DMEM, Gibco), and supplemented with 10%FBS. LNCaP-AI cells were cultured in media with 5% CSS. For the experiments with DHT stimulation, cells were cultured in medium with 5% CSS. Cells were incubated in a humidified incubator containing 5% CO2 at 37°C.

### siRNA and lentiviral production

For RNA interference (RNAi), chemically synthesized small interfering RNAs siRNA duplexes were purchased from Sigma (Sigma-Aldrich). The sequences of siRNAs targeting PRPF6 were: siPRPF6 1# 5'-GAGAAGATTGGGCAGCTTA-3', siPRPF6 2# 5'-GAUCUAAAUGACACCAAUU-3'. The sequence of negative control siRNA (siCtrl) was 5'- UUCUCCGAACGUGUCACGUTT -3'. For lentivirus-delivered RNAi, lentiviral productions targeting the same sequence as siPRPF6 1# were purchased from Shanghai GeneChem Company and performed following manufacturer's instructions.

### Dual-luciferase reporter assays

In dual-luciferase reporter assays, cells were co-transfected with the listed constructs. After 24 hrs, cells harvested and lysed in passive lysis buffer. Luciferase activities were analyzed by dual-luciferase reporter assay system using GloMax Multi Jr detection system (Promega). Firefly luciferase activity was normalized to the activity of control renilla luciferase. Data represent the mean ± SD from three experiments.

### Immunoprecipitation and western blot

For immunoprecipitation, equal protein amounts of the extracted whole cell lysates were immunoprecipitated with specific antibody or control IgG. The immunoprecipitated complexes were analyzed by western blot.

### Immunofluorescence stain

Cells were fixed by paraformaldehyde and blocked in donkey serum albumin. Then cells were incubated with primary antibody and subsequently FITC- or Cy3-conjugated secondary antibody (Jackson ImmunorRsearch Labs Inc). Nuclei were stained with DAPI (Roche).

### RNA isolation and quantitative real-time polymerase chain reaction (real-time PCR)

Total RNA was extracted using TRIzol reagent (Thermo Fisher Scientific). cDNA synthesis was carried out with PrimeScript RT reagent kit with gDNA Eraser (TaKaRa), and then analyzed by SYBR Premix Ex Taq kit (TAKARA) according to the manufacturer's instructions in LightCycler96 system (Roche). Relative expression of genes was normalized by 18S rRNA. Data represent the mean ± SD of triplicate real-time PCR. Sequence of primers were list ed in Supplementary Table S1.

### RNA immunoprecipitation (RIP) and chromatin immunoprecipitation (ChIP)

RIP experiments were performed using control IgG or antibodies using RIP assay kit (Millipore, Magna RIP kit) according to manufacturer's protocol. The immunoprecipitated RNA were converted to cDNA and quantified by real-time PCR. ChIP experiments were performed as previously described 13. The purified immunoprecipitated DNA was quantified by real-time PCR. Results were shown as percentage of input. Data represent the mean ± SD from three experiments. Sequence of primers were listed in Supplementary Table S2.

### Cell growth analysis and colony formation assays

For growth curve assays, cells were seeded into 96-well plates at indicated densities. After indicated times, viability of cells were evaluated using the MTS assay (Promega) with the absorbance at 490 nm. For colony formation assays, cells were maintained in medium for indicated times, then cells were fixed with paraformaldehyde and stained with coomassie blue dye.

### Xenograft tumor growth assays

Animal works were approved and supervised by the Animal Ethics Committee of China Medical University. 5×10^5^cells were suspended in 100ul medium with half Metrigel (BD Biosciences) and injected subcutaneously into the 6-week-old male NOD/SCID mice with or without castration operation. Tumor size were measured by electronic caliper and the volume were calculated according to the formula: Volume=(π/6)×(width×length)^3/2^. 21 or 27 days after inoculation, mice were killed in keeping with the policy of the humane treatment of tumor bearing animals.

### Immunohistochemical (IHC) analysis and clinical prostate cancer samples

Formalin-fixed paraffin-embedded sections of PCa tissues and adjacent non-cancerous tissues were obtained from the first hospital of China Medical University. Ethical approval for sample collection and tissue use was approved by the Human Research Ethics Committee of China Medical University. A total of 162 tissues, including 20 PCa tissues without matched adjacent non-cancerous tissues, and 71 pairs of PCa and matched adjacent non-cancerous tissues, were collected to perform IHC staining. IHC experiments were performed as previously described 12. The immunohistochemical stain of PRPF6 expression was evaluated by the Hscore method, as previously described 26. Briefly, the intensity was scored as follows: 0, negative; 1, weak; 2, moderate and 3, strong. The final score, which ranged from 0 to 3, was determined by summing the intensity score multiplied by the corresponding proportion.

### Statistical analysis

To determinate the significant difference in results of real-time PCR, dual-luciferase reporter assays and xenograft tumor growth assays, Student's *t*-test was used with Prism Graphpad program. SPSS software was used to analyze results of IHC. Student's *t*-test was used to determinate the significant difference between immunohistochemical expression of PRPF6.

## Results

### PRPF6 promotes cell growth in prostate cancer-derived cell lines

**Figure 1:**
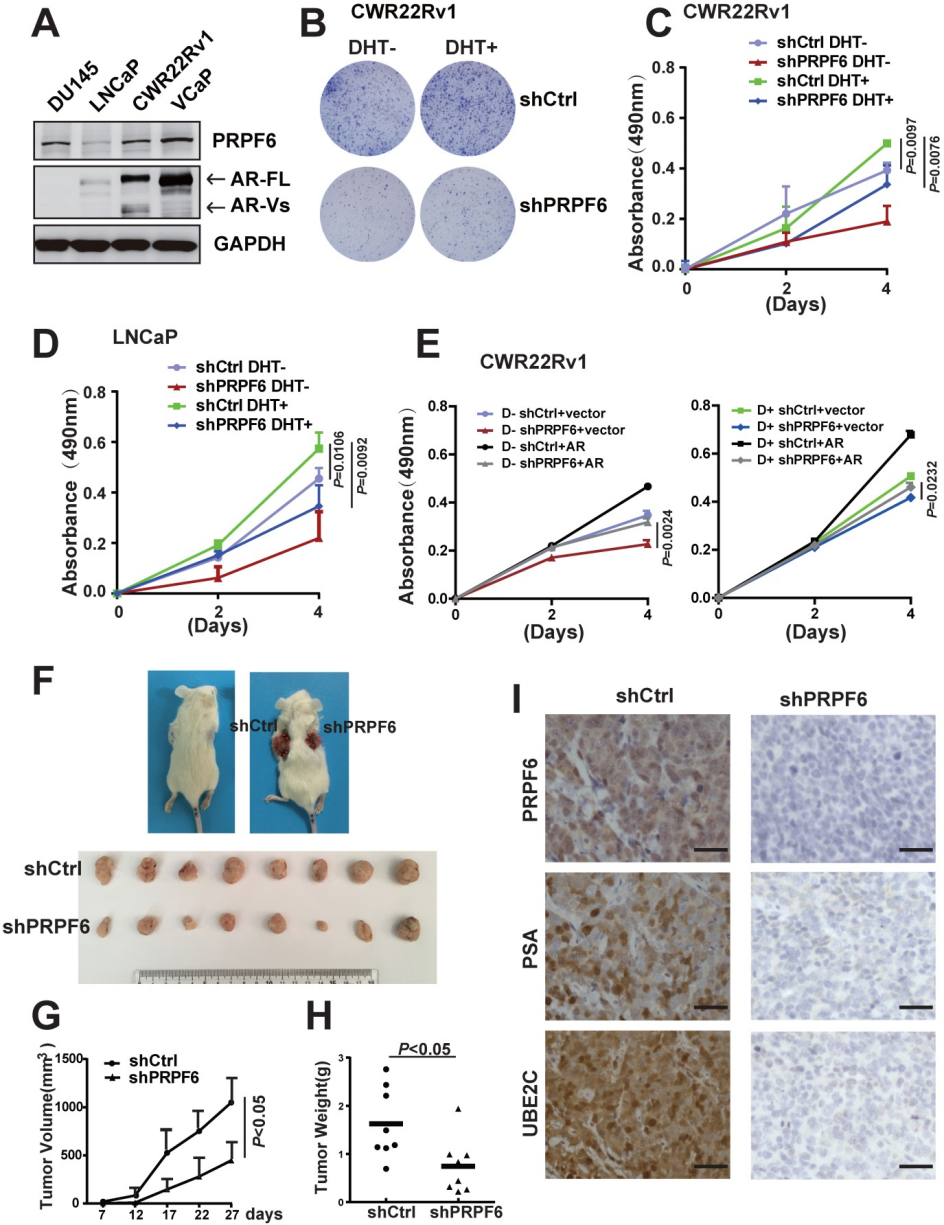
** PRPF6 promotes growth of prostate cancer cells. A.** PRPF6 and AR expressions in prostate cancer-derived cell lines were analyzed by western blot. Protein extractions of different cell lines were subjected to western blot analysis using antibodies against PRPF6 (Proteintech) and AR (Proteintech). GAPDH was used as internal control. **B.** Effects of PRPF6 knockdown on the colony formation of CWR22Rv1 cells were shown. CWR22Rv1 cells (1000 per well) were infected with lentivirus shRNA targeting PRPF6 (shPRPF6) or control lentivirus (shCtrl), and then were treated with 10^-8^ M DHT or ethanol for 14 days. **C and D.** PRPF6 knockdown repressed cell proliferation. CWR22Rv1 cells (C) and LNCaP (D) (2500 per well) with shPRPF6 or shCtrl were treated with 10^-8^ M DHT or ethanol. After indicated time, MTS reagent was added, then absorbance at 490 nm was measured and plotted. Data were means ± SD of three independent experiments. Student's *t*-test was performed. **E.** Growth inhibition of prostate cancer cells by PRPF6 was associated with AR. CWR22Rv1 cells (2500 per well) with shPRPF6 or shCtrl were transfected with AR expression lentivirus or control lentivirus, and then were treated with 10^-8^ M DHT (right) or ethanol (left). After indicated time, MTS reagent was added, then absorbance at 490 nm was measured and plotted. Data were means ± SD of three independent experiments. Student's *t*-test was performed. **F.** Representative photograph of mice with tumor xenografts (upper panel). Photograph of all xenograft tumors derived from CWR22Rv1 cells with shPRPF6 or shCtrl (lower panel). **G.** Tumor volumes of xenografts after inoculation were shown. The volume of xenograft tumors derived from CWR22Rv1 cells with shPRPF6 or shCtrl were measured every 5 days. Data represent means ± SD. Student's *t*-test was performed. **H.** Final tumor weights of xenograft tumors derived from CWR22Rv1 cells with shPRPF6 or shCtrl were measured and shown. Horizontal lines represent the mean values. Student's *t*-test was performed. **I.** Expressions of indicated proteins in xenograft tumors were detected by IHC assays. Scale bars, 100 µm.

To investigate the physiological role of PRFP6 in prostate cancer, we examined the endogenous protein expressions of PRPF6 in four cultured prostate cancer-derived cell lines as indicated (Figure 1A). These cells generally expressed PRPF6 protein. In three AR-positive prostate cancer cell lines, PRPF6 was lowly expressed in LNCaP cells that expressed AR-FL but not AR-Vs. In CWR22Rv1 cells and VCaP cells, which constitutively carry AR-FL and AR-Vs, PRPF6 were significantly highly expressed. As we previously reported that PRPF6 enhances transactivation function of AR 25, we then analyzed the impact of PRPF6 on the growth and motility characteristics of AR-positive prostate cancer cells by lentivirus-mediated knockdown of PRPF6 (Supplementary Figure S1). Colony formation experiments were performed in the absence and presence of androgen (DHT), and results showed PRFP6 knockdown impaired cell ability to form colonies in CWR22Rv1 cells (Figure 1B). The same trend was showed in LNCaP cells with lower ability of colonies formation (Supplementary Figure S2). Consistently, cell growth with PRFP6 knockdown was slower than control cells, in both CWR22Rv1 (Figure 1C) and LNCaP cells (Figure 1D). Further, exogenous expression of AR rescued the growth inhibition by PRPF6 knockdown in CWR22Rv1 cells, indicating that growth promotion by PRPF6 is associated with AR (Figure 1E).

In addition, we performed *in vivo* tumor growth analysis in a mouse xenograft model with CWR22Rv1 cells. Results showed that PRPF6 knockdown reduced tumor burden, with smaller volumes and slower growth rate of the xenograft tumors (Figure 1F and 1G). In line with the tumor growth curve, the tumor weights of cells with PRPF6 knockdown were significantly lower (Figure 1H). Consistent with the AR-related growth promoting function of PRPF6, xenograft tumors derived by PRPF6 knockdown cells exhibited a significant reduction in prostate-specific antigen (PSA) and ubiquitin-conjugating enzyme E2 C (UBE2C) levels, which are target genes of AR (Figure 1I). Taken together, the above results indicated that PRPF6 promotes cell proliferation of prostate cancer cells *in vitro* and *in vivo*.

### PRPF6 associates with AR in prostate cancer cells

**Figure 2:**
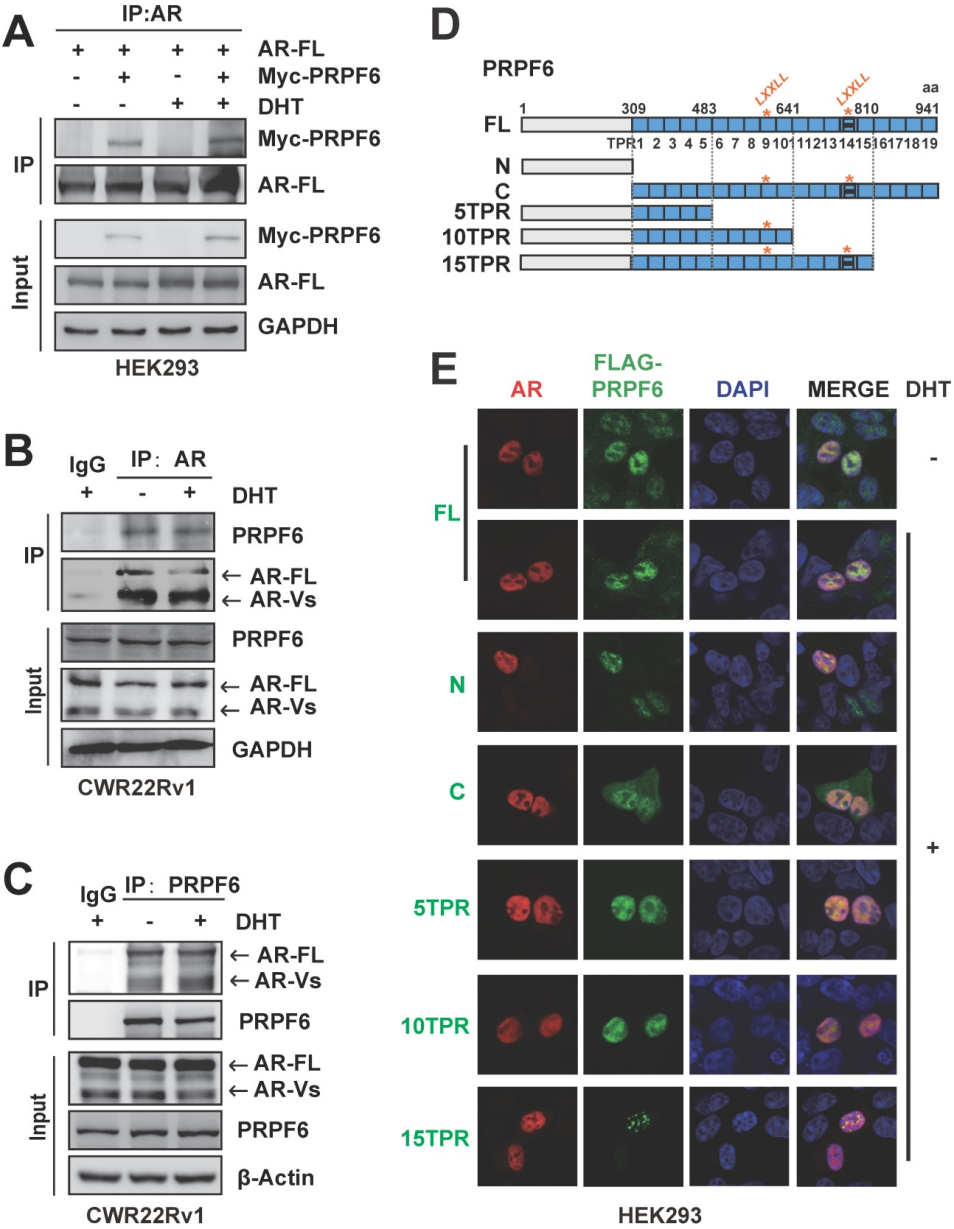
** PRPF6 interacts with AR in prostate cancer cells. A.** Exogenous PRPF6 interacted with AR-FL in HEK293 cells. HEK293 cells were co-transfected with plasmids expressing AR-FL and PRPF6 or vector as indicated. After 4 hrs, cells were treated by DHT (10^-8^ M) or ethanol vehicle for another 24 hrs. Then cells were harvested and equal amounts of cell lysates were subjected to immunoprecipitation with anti-AR antibody (Invitrogen). The immunoprecipitated proteins were subjected to western blot analysis using anti-AR (Proteintech) and the indicated antibodies. GAPDH was used as internal control. **B and C.** Endogenous PRPF6 and AR interacted with each other in CWR22Rv1 cells. Upon treatment of 10^-8^ M DHT or ethanol vehicle for 24 hrs, CWR22Rv1 cells were harvested and equal amounts of cell lysates were subjected to immunoprecipitation with normal IgG, anti-AR antibody (Invitrogen) or anti-PRPF6 antibody (Santa Cruz). The immunoprecipitated proteins were subjected to western blot analysis using antibodies against PRPF6 (Proteintech) and AR (Proteintech). GAPDH or β-Actin was used as internal control. **D.** Diagram of plasmids encoding FLAG-tagged PRPF6-FL and truncated mutants. **E.** Subcellular localizations of the AR and PRPF6-FL or truncated mutants. HEK293 cells were co-transfected with plasmids expressing AR-FL and PRPF6-FL or truncated mutants. After 10^-8^ M DHT stimulation or ethanol vehicle for 4 hrs, cells were fixed and stained with antibody against FLAG and AR (Proteintech). DAPI was used to visualize the nucleus (blue). Merged images were shown as indicated.

We then wonder to test the association between PRPF6 and AR in prostate cancer cells. Co-IP results showed that exogenous expressed AR-FL was precipitated with Myc-tagged PRPF6 in HEK293 cells, both in the absence and presence of DHT (Figure 2A). Further, in CWR22Rv1 cells, the interaction between endogenous PRPF6 and AR-FL could be observed (Figure 2B and 2C). In addition to AR-FL, PRPF6 also interacted with endogenous AR-Vs in CWR22Rv1 cells (Figure 2C). Moreover, results of immunofluorescence experiments showed that endogenous PRPF6 located in the nucleus in prostate cancer cells including LNCaP, CWR22Rv1, and DU145 (Supplementary Figure S3A-S3C). In LNCaP cells, PRPF6 was compartmentalized in the nucleus with AR under DHT treatment (Supplementary Figure S3A). In CWR22Rv1 cells, PRPF6 was distributed together with AR in the nucleus, both in the absence and presence of DHT (Supplementary Figure S3B). These data indicated that PRPF6 physically associates with AR in prostate cancer cells.

PRPF6 protein contains an N-terminal domain followed by multiple tetratricopeptide repeat (TPR) motifs. In addition, we generated expression plasmids encoding PRPF6 truncated mutants (Figure 2D and Supplementary Figure S4). Our results of immunofluorescence experiments showed that PRPF6 C (309-941 aa) localized in both nucleus and cytoplasm (Figure 2E). Meanwhile, PRPF6 N (1-308 aa), PRPF6 full length (FL), PRPF6 5TPR (1-482 aa), PRPF6 10TPR (1-640 aa) and PRPF6 15TPR (1-809 aa) showed nuclear localization (Figure 2E). And the localizations of PRPF6 5TPR (1-482 aa) and PRPF6 10TPR (1-640 aa) were almost identical to PRPF6 FL, whereas PRPF6 15TPR (1-809 aa) showed obvious speckle localization (Figure 2E).

### PRPF6 coactivates AR-FL or AR-V7-mediated transactivation in prostate cancer cells

**Figure 3:**
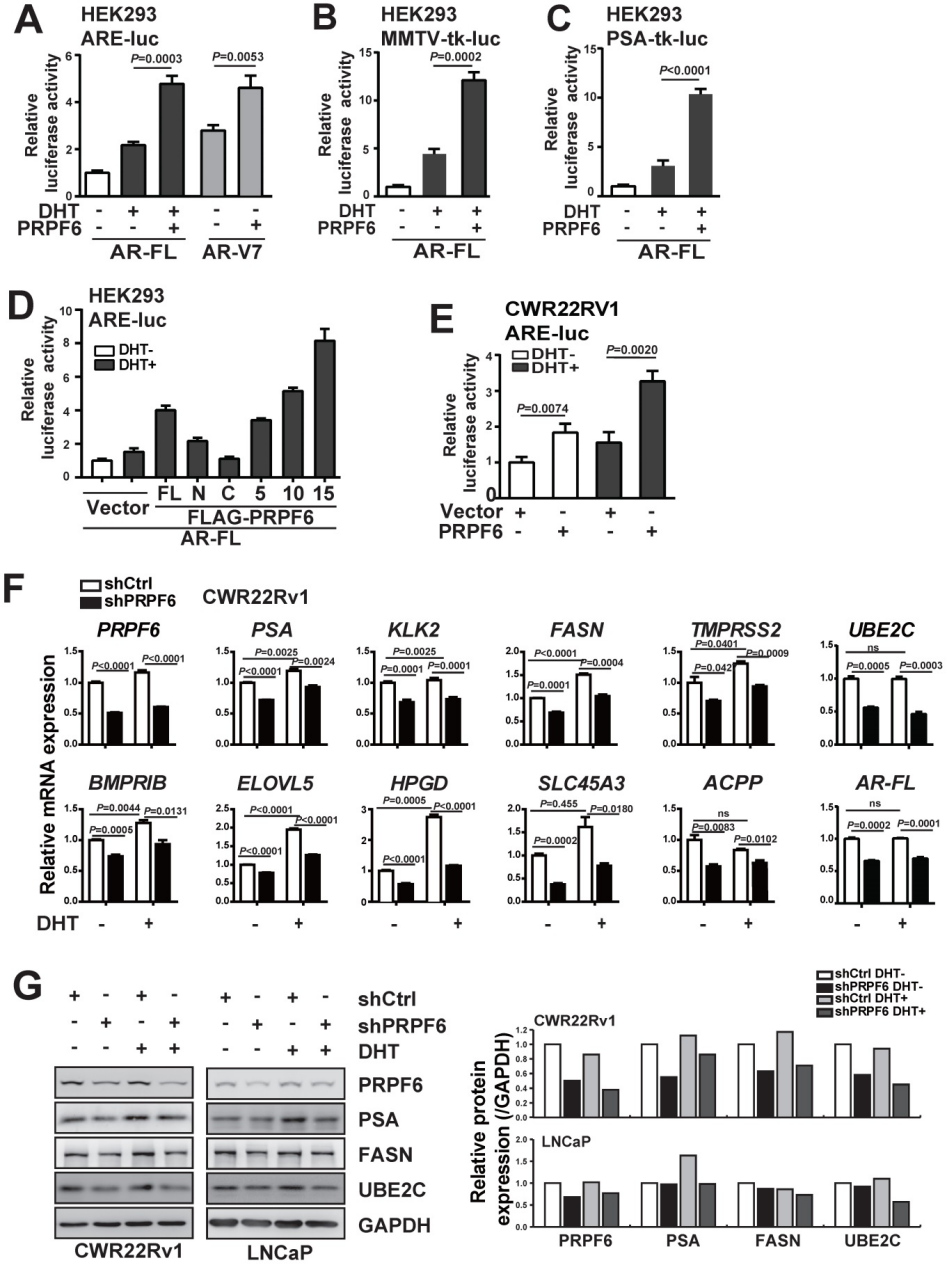
** PRPF6 promotes AR-FL and AR-V7-mediated transactivation. A, B and C.** PRPF6 enhanced AR-FL and AR-V7-mediated transactivation. HEK293 cells were co-transfected with AR-FL (A, B and C) or AR-V7 (A) and pRL-TK, together with the indicated reporter luciferase plasmids. After 4 hrs, cells were treated by DHT (10^-8^ M) or ethanol vehicle for another 24 hrs. Then, cells were lysed and assayed using the dual-luciferase reporter assay system. Student's *t*-test was performed. **D.** Effects of PRPF6-FL or truncated mutants on AR-FL-mediated transactivation. HEK293 cells were co-transfected with and ARE-luc, pRL-TK, together with AR-FL and indicated plasmids. After 10^-8^ M DHT stimulation or ethanol vehicle for 24 hrs, cells were lysed and assayed using the dual-luciferase reporter assay system. **E.** PRPF6 enhanced endogenous AR-mediated transactivation in CWR22Rv1 cells. CWR22Rv1 cells were co-transfected with and ARE-luc, pRL-TK, together with PRPF6 or vector plasmids as indicated. After 4 hrs, cells were treated by DHT (10^-8^ M) or ethanol vehicle for another 24 hrs. Then, cells were lysed and assayed using the dual-luciferase reporter assay system. Student's *t*-test was performed. **F.** Effect of PRPF6 knockdown on the mRNA expression of AR target genes and* AR-FL* in CWR22Rv1 cells. Cells were infected with shPRPF6 or shCtrl. After treatment of 10^-8^ M DHT or ethanol vehicle for 24 hrs, cells were collected for RNA extraction and quantitative real-time PCR were performed. Student's *t*-test was performed. ns, not significant. **G.** Effect of PRPF6 knockdown on the protein expression of AR target genes in CWR22Rv1 and LNCaP cells. CWR22Rv1 (left) or LNCaP (right) cells with shPRPF6 or shCtrl were treated with 10^-8^ M DHT or ethanol vehicle for 24 hrs, then cells were harvested for protein extraction. Western blot was performed to detect the expression of proteins using anti-PRPF6 (Santa Cruz) and the indicated antibodies. GAPDH was used as internal control. The protein expressions were quantified by densitometry and shown in the right panel, with GAPDH as the reference.

We next performed a series of luciferase assays to investigate the function of PRPF6 on AR-mediated transcription. By employing three luciferase reporter systems, including ARE-luc, MMTV-tk-luc and PSA-tk-luc reporters, we demonstrated that PRPF6 significantly enhanced AR-FL-mediated transactivation in the presence of DHT (Figure 3A-3C). Further, PRPF6 significantly up-regulated AR-V7-mediated transcriptional activity in the absence of DHT (Figure 3A). Moreover, luciferase assays were performed with these truncated mutants. PRPF6 5TPR and PRPF6 10TPR showed similar ability to enhance AR-FL-mediated transactivation as PRPF6 FL, while nor PRPF6 N or PRPF6 C has obvious effect (Figure 3D). Intriguingly, PRPF6 15TPR showed significant enhancement of AR-FL-mediated transactivation (Figure 3D). In CWR22Rv1 cells, PRPF6 significantly enhanced the transcriptional activity of ARE-luc reporter gene with or without DHT treatment, which represents the endogenous AR-FL and AR-Vs-mediated transactivation (Figure 3E). Based on the above results, we conclude that PRPF6 functions as a coactivator of AR-FL, and enhances AR-V7 action in the absence of DHT.

Having demonstrated that PRPF6 functions as a cofactor of AR-FL and AR-V7, we next examined whether PRPF6 knockdown led to altered expression of endogenous AR target genes. To this end, we first turn to LNCaP cells, which is androgen-sensitive and without AR-V7 expression. We evaluated the mRNA expression of a panel of well-characterized AR targets, that include *PSA*, *kallikrein related peptidase 2 (KLK2*), *fatty acid synthase* (*FASN*), *transmembrane protease serine 2* (*TMPRSS2*),* UBE2C*, *bone morphogenetic protein type IB receptor* (*BMPRIB*), *ELOVL fatty acid elongase 5* (*ELOVL5*), *15‐hydroxyprostaglandin dehydrogenase* (*HPGD*), *solute carrier family 45 a3 member* (*SLC45A3*), and *acid phosphatase 3* (*ACPP*). Rea-time PCR results showed that lentivirus-mediated PRPF6 knockdown resulted in a significant decrease in the expression of 9 AR target genes, including *PSA*, *KLK2*, *FASN*, *TMPRSS2*, *UBE2C*, *BMPRIB*, *HPGD*, *SLC45A3* and *ACPP* with DHT treatment in LNCaP cells (Supplementary Figure S5). Meanwhile, we analyzed the impact of PRPF6 knockdown in CWR22Rv1 cells, and found expressions of the 10 genes were all repressed both with and without DHT treatment (Figure 3F). Interestingly, PRPF6 knockdown suppressed expression of *AR-FL* (Supplementary Figure S5 and Figure 3F). Moreover, western blot analysis of the endogenous protein expression in LNCaP cells confirmed that knockdown of PRPF6 led to obvious reductions of PSA protein levels under DHT treatment, while repressed FASN and UBE2C protein expressions both with and without DHT treatment (Figure 3G). In CWR22Rv1 cells, PSA, FASN and UBE2C protein expressions were downregulated by PRPF6 knockdown in the absence or presence of DHT, respectively (Figure 3G). Taken together, these data suggested that PRPF6, as a cofactor, is required for endogenous AR-regulated gene activation.

### PRPF6 associates with JMJD1A to enhance AR-induced transactivation

**Figure 4:**
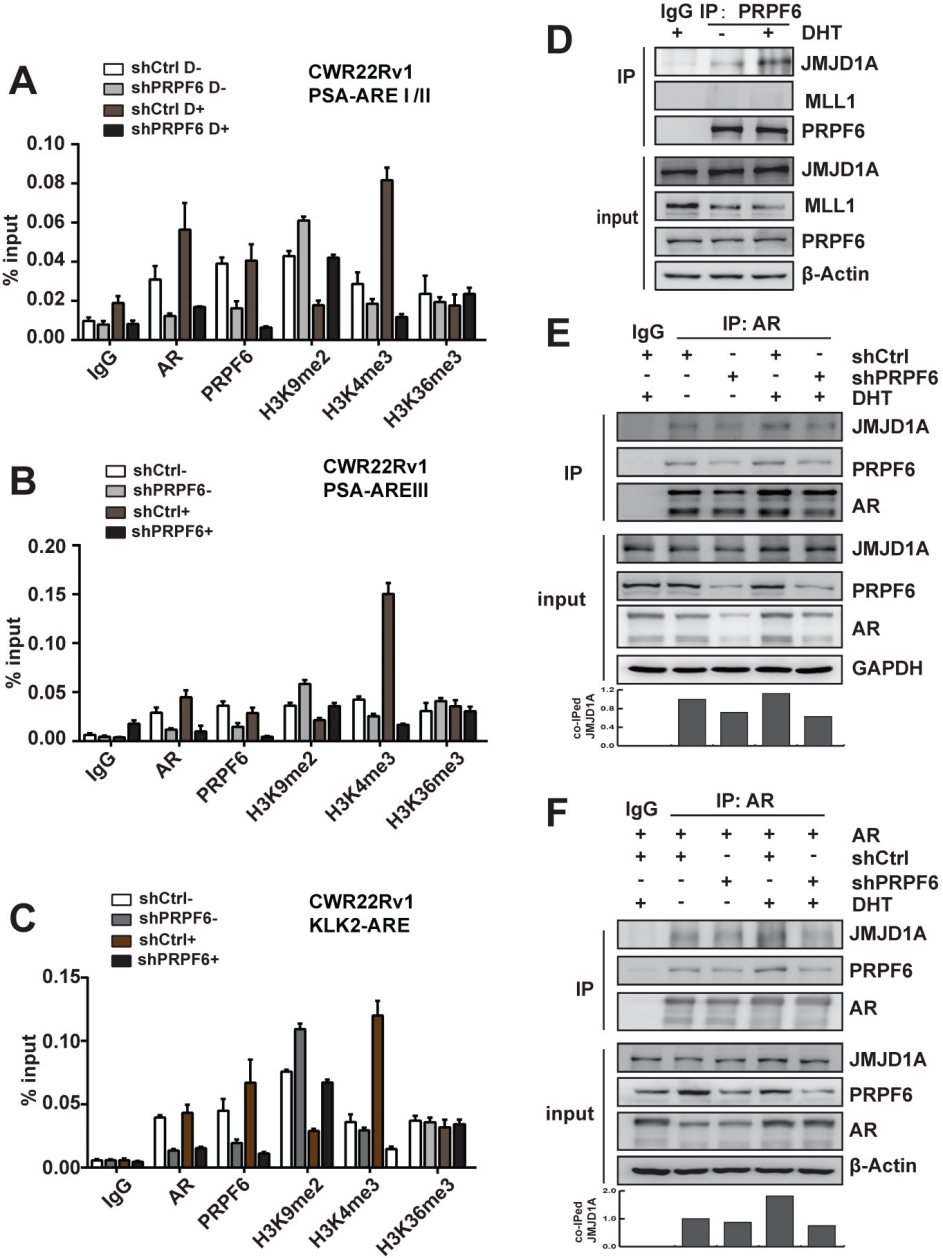
** Mechanisms involved in promotion of AR-mediated transactivation by PRPF6. A, B and C.** PRPF6 knockdown impacted on the recruitments of PRPF6 and AR, as well as the epigenetic modification on AREI/II (A), AREIII (B) in *PSA* and ARE (C) in *KLK2*. CWR22Rv1 cells infected with shPRPF6 or shCtrl were treated with 10^-8^ M DHT or ethanol for 4 hrs, then cells were subjected to ChIP assays with using normal IgG, anti-AR (Invitrogen), anti-PRPF6 (Bethyl) or the indicated antibodies. **D.** PRPF6 interacts with JMJD1A in CWR22Rv1 cells. Upon treatment of 10^-8^ M DHT or ethanol vehicle for 24 hrs, CWR22Rv1 cells were harvested and equal amounts of cell lysates were subjected to immunoprecipitation with normal IgG, anti-PRPF6 antibody (Proteintech). The immunoprecipitated proteins were subjected to western blot analysis using antibodies against PRPF6 (Proteintech), MLL1 (Bethyl) and JMJD1A (Proteintech). β-Actin was used as internal control. **E and F.** PRPF6 knockdown reduced the interaction between JMJD1A and AR. CWR22Rv1 cells infected with shPRPF6 or shCtrl were transfected with AR expression plasmid (in F) or not (in E). Then, upon treatment of 10^-8^ M DHT or ethanol vehicle for 24 hrs, cells were harvested and equal amounts of cell lysates were subjected to immunoprecipitation with normal IgG, anti-AR antibody (Invitrogen). The immunoprecipitated proteins were subjected to western blot analysis using antibodies against PRPF6 (Proteintech), JMJD1A (Proteintech) and AR (Proteintech). GAPDH was used as internal control. Co-immunoprecipitated (co-IPed) JMJD1A was quantified by densitometry and shown in the lower panel.

We next investigated molecular mechanisms underlying the modulation of PRPF6 on AR-mediated transcription. AR binds to cognate AR-responsive elements (AREs) on its target genes to induce target gene transcription upon the treatment of androgen. We thus turn to examine the recruitment of PRPF6 or AR on the *cis*-regulatory elements of target genes with ChIP assay. The results demonstrated that AR or PRPF6 was recruited to AREI/II region in the promoter of *PSA* gene (Figure 4A). A growing body of evidence support that coordination of pre-mRNA splicing with chromatin remodeling events are involved in transcriptional regulation 27-29, we then ask whether PRPF6 would influence some epigenetic events, including histone modification. Histone H3K4me and H3K36me are hallmarks of actively transcribed euchromatin, whereas H3K9me is associated with repressive heterochromatin state. We then tested the alteration of histone H3K9me2, H3K4me3, or H3K36me3 at chromatin encompassing the *cis*-regulatory elements in the cells with knockdown of PRPF6. Interestingly, our results showed that PRPF6 depletion increased histone H3K9me2 level and decreased H3K4me3 level, while alternation of histone H3K36me3 was not obvious (Figure 4A). Similar results were observed at ARE III region in *PSA* enhancer and ARE region in *KLK2* promoter (Figure 4B and 4C). In addition, PRPF6 knockdown also decreased the recruitment of AR at the examined sites (Figure 4A-4C).

We then turn to ask whether PRPF6 associates with histone methyltransferase or demethylase to coactivate AR-mediated transactivation. It has been reported that JMJD1A, which is a histone H3K9 demethylase, acts as a coactivator for AR by epigenetic regulation of histone H3K9me marks 30, 31. The mixed-lineage leukemia 1 (MLL1), which is a histone methyltransferase of H3K4, could be recruited at ARE of AR target genes, subsequently increasing histone H3K4 trimethylation 13. In order to determine whether PRPF6 might interact with JMJD1A or MLL1 to regulate AR-mediated transcription, Co-IP experiments were performed to examine the association of PRPF6 with JMJD1A or MLL1. The results demonstrated an interaction between PRPF6 and JMJD1A, but not MLL1 (Figure 4D). Moreover, AR interacts with PRPF6 together with JMJD1A, and knockdown of PRPF6 weakened the interaction between JMJD1A and AR (Figure 4E), which is in line with the alternation of histone H3K9me2 at ARE regions by PRPF6 knockdown. Since PRPF6 knockdown can reduce the expression of endogenous AR, we further detected the interaction between JMJD1A and AR under AR overexpressing condition. Similar to the result showed in Figure 4E, Co-IP experiment showed decreased interaction between JMJD1A and AR by PRPF6 knockdown (Figure 4F). Taken together, we speculate that PRPF6 associates with JMJD1A, thereby altering the histone H3K9me2 level at the ARE regions of *AR* target genes to maintain the active chromatin state, subsequently promoting AR-mediated transcription.

### PRPF6 knockdown attenuates AR and AR-V7 transcription

**Figure 5:**
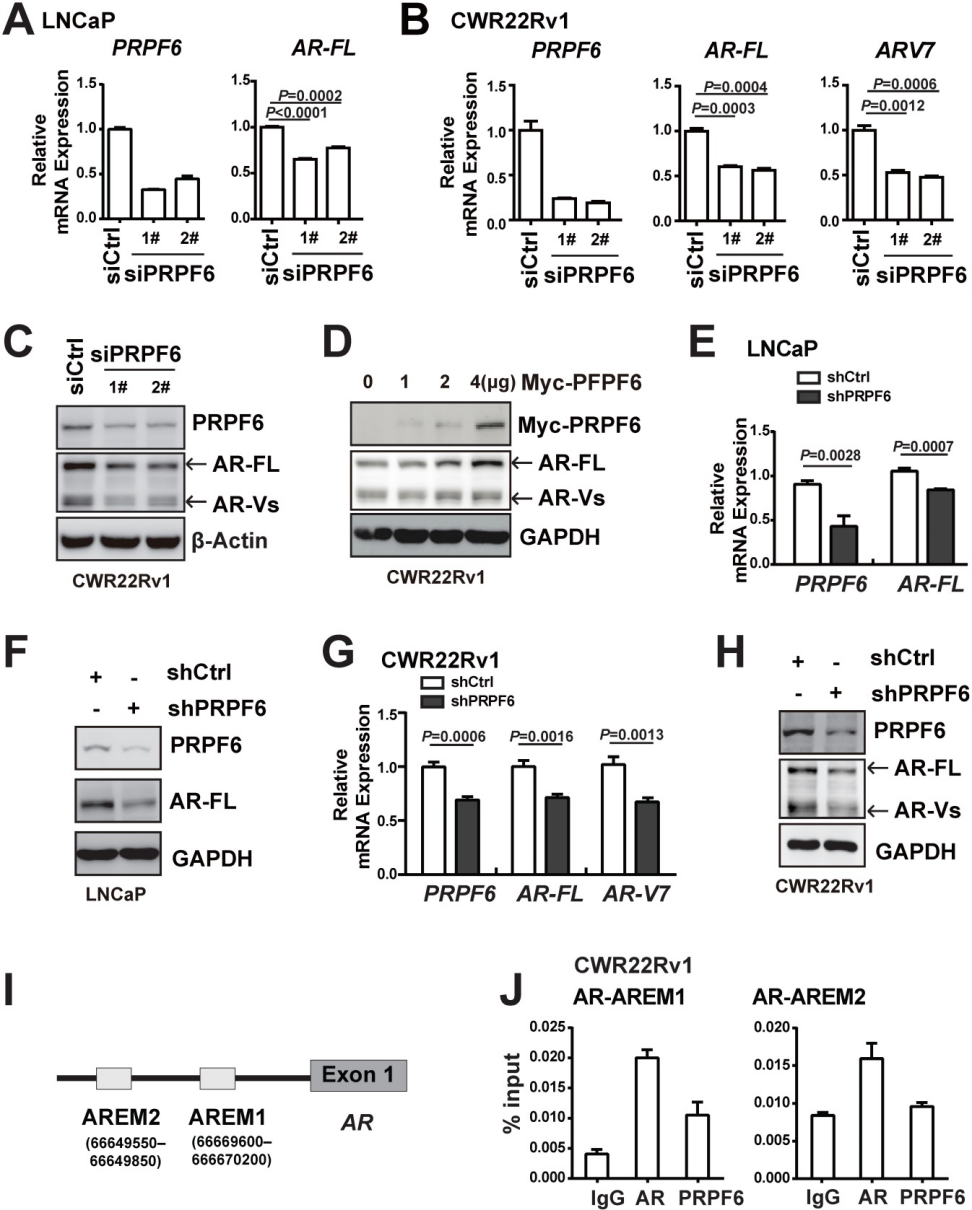
** PRPF6 promotes the expressions of AR-FL and AR-V7. A and B.** Knockdown of PRPF6 downregulated mRNA expressions of *AR-FL* in LNCaP cells (A), and downregulated mRNA expression of *AR-FL* and* AR-V7* in CWR22Rv1 cells (B). Cells were transfected with two independent siRNAs against PRPF6 (siPRPF6) or control siRNA (siCtrl). After 48 hrs, cells were collected for RNA extraction and real-time PCR were performed. Student's *t*-test was performed. **C.** Knockdown of PRPF6 downregulated protein expressions of AR-FL and AR-Vs in CWR22Rv1 cells. CWR22Rv1 cells were transfected with siCtrl or siPRPF6. After 48 hrs, cells were collected for protein extraction and subjected to western blot analysis using antibodies against PRPF6 (Santa Cruz) and AR (Proteintech). β-Actin was used as internal control. **D.** Ectopic expression of PRPF6 upregulated protein expressions of AR-FL and AR-Vs in CWR22Rv1 cells. CWR22Rv1 cells were transfected with different amounts of Myc-tagged PRPF6 expression plasmids or control vector. After 48 hrs, cells were collected for protein extraction and subjected to western blot analysis using anti-AR (Proteintech) and the indicated antibodies. GAPDH was used as internal control. **E and F.** Impacts of lentivirus-mediated PRPF6 knockdown on the expression of AR-FL in LNCaP cells. LNCaP cells with shPRPF6 or shCtrl were collected for RNA extraction and real-time PCR were performed (E), or protein extraction and subjected to western blot analysis using antibodies against PRPF6 (Santa Cruz) and AR (Proteintech) (F). **G.** Impacts of lentivirus-mediated PRPF6 knockdown on the mRNA expressions of *AR-FL* and *AR-V7* in CWR22Rv1 cell. CWR22Rv1 cell with shPRPF6 or shCtrl were collected for RNA extraction and subjected to real-time PCR. **H.** Impacts of lentivirus-mediated PRPF6 knockdown on the protein expressions of AR-FL and AR-Vs in CWR22Rv1 cells. Cells were infected with shPRPF6 or shCtrl, and then were collected for protein extraction and subjected to western blot analysis using antibodies against PRPF6 (Santa Cruz) and AR (Proteintech). GAPDH was used as internal control. **I.** Schematic represents AR binding cis-elements in *AR* gene. **J.** PRPF6 was recruited to AR-binding cis-elements in *AR* gene. CWR22Rv1 cells were subjected to ChIP assays using normal IgG, anti-AR (Invitrogen) or anti-PRPF6 (Bethyl).

It is noticeable that PRPF6 knockdown reduced the mRNA (Figure 3F and Supplementary Figure S5) and protein expression of AR (Figure 4E), indicating that PRPF6 might be involved in *AR* itself transcription. In order to assess the effect of PRPF6 on endogenous expression of AR, we knocked down PRPF6 expression using two specific siRNAs targeting different sequences of *PRPF6*. In LNCaP cells, mRNA level of *AR-FL* was reduced after PRPF6 knockdown (Figure 5A). In CWR22Rv1 cells, PRPF6 knockdown resulted in decreased mRNA levels of both *AR-FL* and *AR-V7* (Figure 5B), which is the major form of AR-Vs in this cell line 32. Similarly, knockdown of PRPF6 decreased the protein levels of both AR-FL and AR-Vs (Figure 5C), and ectopic expression of PRPF6 increased the protein expressions of both AR-FL and AR-Vs (Figure 5D) in CWR22Rv1 cells. Consistent with these results, both mRNA and protein levels of AR-FL as well as AR-V7 were significantly reduced upon lentivirus-mediated PRPF6 knockdown (Figure 5E-5H). We next assessed whether PRPF6 participates in alternative splicing of *AR-V7* in CWR22Rv1 cells. As shown in Supplementary Figure S6A, constitutively expressed *AR-FL* mRNA consists of eight exons, whereas *AR-V7* mRNA only includes exons 1-3 and a cryptic exon 3b. We performed RIP experiment to identify potential PRPF6-binding regions using primers targeting the P3b region 32, and the results showed strong enrichment of PRPF6 on P3b region (Supplementary Figure S6B), indicating that PRPF6 may participates in AR-V7 alternative splicing.

It is reported that AR could sustain its own expression by binding enhancers located upstream of the transcription start site in the absence of ligand 33. We therefore evaluated PRPF6 recruitment on the reported AREM1 and AREM2 sites (Figure 5I) by ChIP assay using site-specific primers. Our data showed that PRPF6 was recruited at AREM1 site (Figure 5J), while AR was recruited at both AREM1 and AREM2 sites as reported, suggesting that PRPF6 may be involved in modulation of AR gene transcription. Taken together, these data indicated that PRPF6 promotes AR-FL and AR-V7 transcription and may play a role in transactivation of *AR* gene.

### PRPF6 is required for growth of prostate cancer cells under androgen-depleted condition

**Figure 6:**
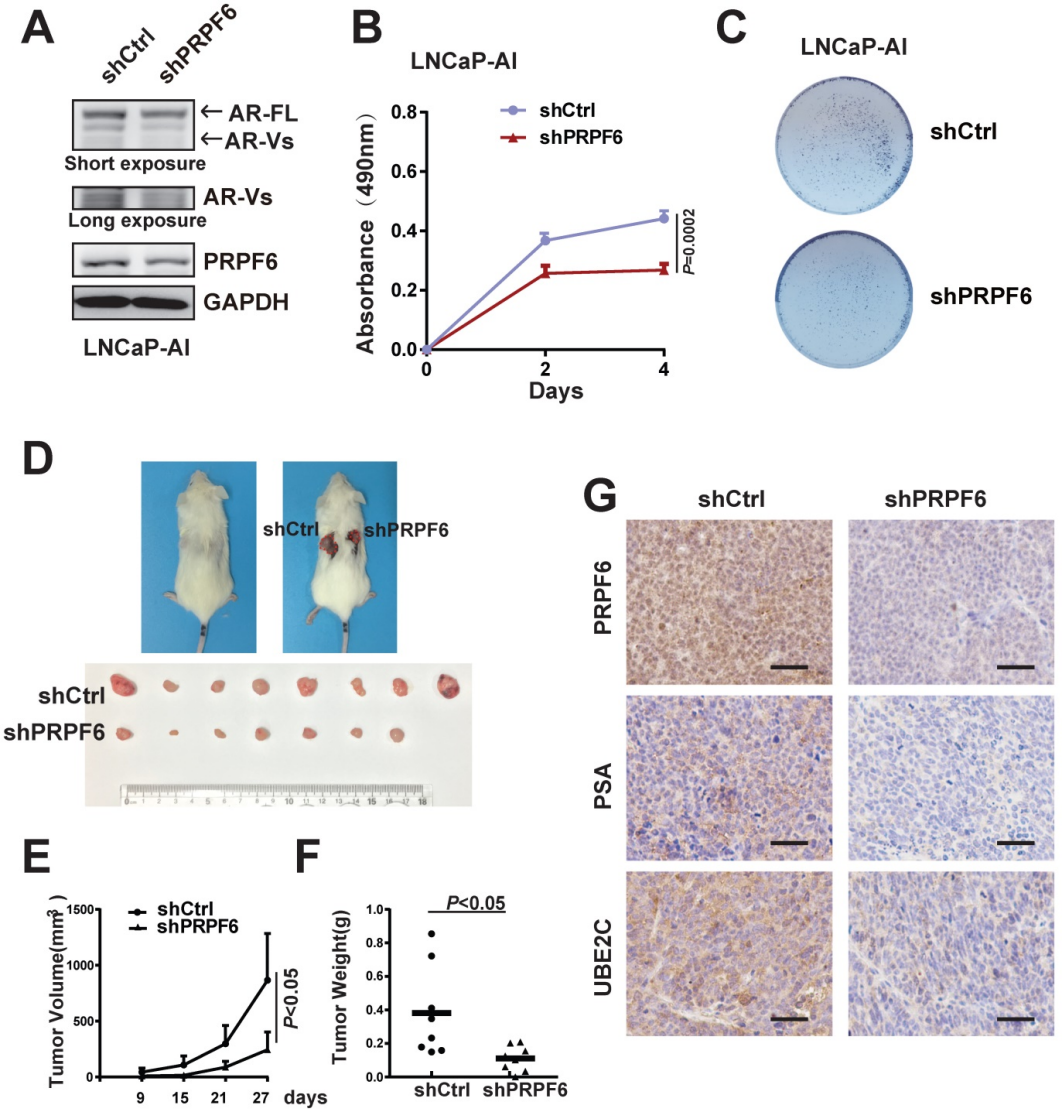
** PRPF6 promotes cell growth under androgen-depleted condition. A.** Impacts of lentivirus-mediated PRPF6 knockdown on the protein expressions in LNCaP-AI cells. LNCaP-AI cells were infected with shPRPF6 or shCtrl, and then were collected for protein extraction and subjected to western blot analysis using antibodies using anti-PRPF6 (Proteintech), anti-AR (Proteintech) and GAPDH. **B.** PRPF6 knockdown repressed cell proliferation of LNCaP-AI cells. LNCaP-AI cells (2500 per well) with shPRPF6 or shCtrl were cultured in medium with 5% CSS. After indicated time, MTS reagent was added, then absorbance at 490 nm was measured and plotted. Data were means ± SD of three independent experiments. Student's *t*-test was performed. **C.** PRPF6 knockdown inhibited cell growth of LNCaP-AI cells under androgen-depleted condition. LNCaP-AI cells (5000 pre well) with shPRPF6 or shCtrl were infected with shPRPF6 or shCtrl, and then were cultured for 14 days. **D.** PRPF6 knockdown inhibited cell growth in xenograft mice under castration condition. Castration operation was performed on 5-week-old male NOD/SCID mice. After one week, CWR22Rv1 cells with shPRPF6 or shCtrl were injected subcutaneously into castrated male mice. Representative photograph of mice with tumor xenografts (upper panel). Photograph of all xenograft tumors derived from CWR22Rv1 cells with shPRPF6 or shCtrl (lower panel). **E.** Tumor volumes of xenografts in castrated male mice were shown. The volume of xenograft tumors derived from CWR22Rv1 cells with shPRPF6 or shCtrl were measured every 6 days. Data represent means ± SD. Student's *t*-test was performed. **F.** Final tumor weights of xenograft tumors derived from CWR22Rv1 cells with shPRPF6 or shCtrl in castrated male mice were measured and shown. Horizontal lines represent the mean values. Student's *t*-test was performed. **G.** Expressions of indicated proteins in xenograft tumors were detected by IHC assays. Scale bars, 100 µm.

Both AR-FL and AR-V7 are necessary to support proliferation of CRPC cells under castration conditions 34. We then set up to test whether PRPF6 is necessary for prostate cancer cell growth under androgen-depleted condition. We first investigated the PRPF6 function in long-term androgen deprivation cells. We generated an androgen-independent LNCaP subline (named LNCaP-AI) by culturing LNCaP cells in media containing androgen-deprived charcoal/dextran-stripped serum (CSS) (5%), which mimics androgen deprivation conditions, for about 6 months. The LNCaP-AI cells were able to proliferate under CSS (Supplementary Figure S7A) and showed a higher ability for colony formation (Supplementary Figure S7B). Similar to previous description 35, LNCaP-AI cells exhibited significant increased expression of AR-FL and AR-V7 (Supplementary Figure S7C and S7D). Consistent with the results in CWR22Rv1 cells, decreased expressions of AR-FL and AR-Vs after PRPF6 knockdown were observed in the LNCaP-AI cells (Figure 6A). In addition, knockdown of PFPF6 reduced colony formation and growth of LNCaP-AI cells under CSS culture conditions (Figure 6B and 6C).

To further validate the role of PRPF6 in tumor growth under castration conditions, we next performed *in vivo* xenograft assays in castrated male mice. As shown in Figure 6D, tumor formation from castration-resistant CWR22Rv1 cells was observed in castrated male mice, and knockdown of PRPF6 significantly suppressed xenograft tumor growth derived from CWR22Rv1 cells. Compared with control cells, PRPF6-knockdown cells showed slower growth rate, smaller volume and decreased tumor weight (Figure 6E and 6F). We also examined the staining of PSA and UBE2C in xenograft tumors, and the result showed that PSA and UBE2C levels were decreased in PRPF6 knockdown cell-derived xenograft tumors (Figure 6G). Taken together, these data suggested that PRPF6 promotes prostate cancer cell growth under androgen-depleted condition *in vitro* and *in vivo*.

### PRPF6 is highly expressed in clinical prostate cancer samples

**Figure 7:**
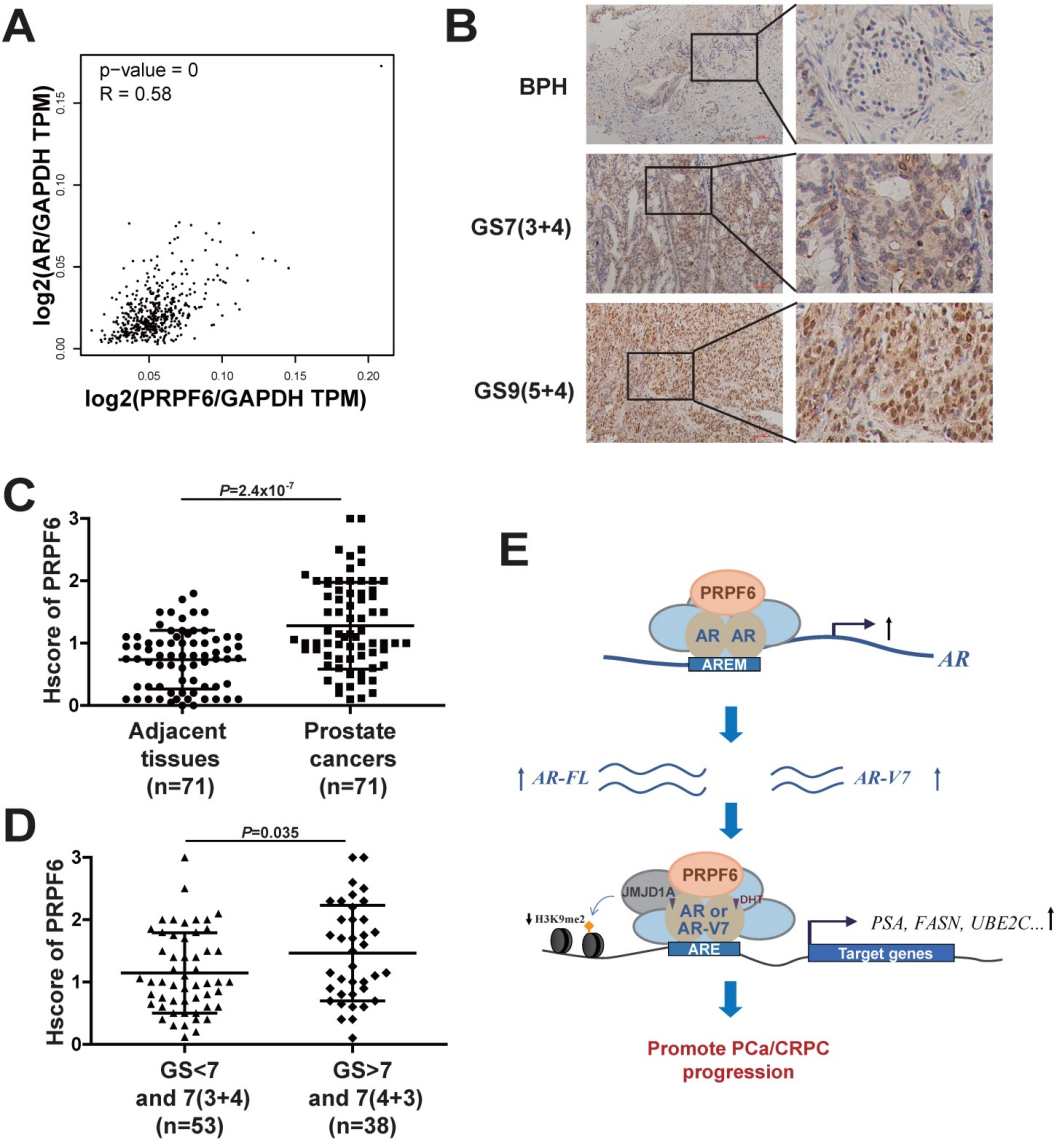
** PRPF6 expressions in human prostate cancers and adjacent noncancerous tissues. A.** Pearson correlation between mRNA expression of *PRPF6* and that of *AR* in prostate cancer from TCGA database. The relative level of *PRPF6* normalized by GAPDH was plotted against that of *AR* via GEPIA (http://gepia.cancer-pku.cn/). **B.** Representative images of PRPF6 immunohistochemical staining in human prostate cancers and adjacent noncancerous tissues. BPH, benign prostate hyperplasia. GS, Gleason score. Scale bars, 100 µm. **C.** Increased expression of PRPF6 in clinical prostate cancer tissues, compared with the matched adjacent non-cancerous tissues. Horizontal lines represent the means ± SD. Student's *t*-test was performed. **D.** PRPF6 expression in prostate cancer samples with different Gleason score. Horizontal lines represent the means ± SD. Student's *t*-test was performed. **E.** Schematic representation of the functions of PRPF6 in prostate cancer and CRPC.

Having revealed the involvement of PRPF6 in AR signaling and growth of prostate cancer cells, we next examined the expression of PRPF6 in clinical prostate cancer samples. In good agreement with upregulation of AR-FL and AR-Vs expression by PRPF6 (Figure 5), we found that mRNA expression of *PRPF6* was positively correlated with that of *AR* in prostate cancer patients of TCGA database using the Gene Expression Profiling Interactive Analysis (GEPIA) 36, suggesting PRPF6 participate in regulation of *AR* transcription in clinical prostate cancer tissues (Figure 7A). We then examined the expression of PRPF6 in 71 pairs of prostate cancer tissues and the matched adjacent noncancerous tissues by immunohistochemical stain (IHC). The results showed that PRPF6 primarily localized in nucleus, as well as mild cytoplasmic staining was also observed (Figure 7B). The stain intensity of PRPF6 was increased in prostate cancer tissues compared with the matched adjacent noncancerous tissues (Figure 7C). Further, in the above 71 prostate cancer tissues and another 20 prostate cancer tissues, PRPF6 expression was higher in prostate cancers with higher Gleason scores (Figure 7D). These results suggested that PRPF6 might play a role in promotion of clinical prostate cancers.

## Discussion

It has been demonstrated that several splicing factors are upregulated and drives the progression of prostate cancer 5, 6. In this study, we found that PRPF6 is recruited to *cis*-response elements on AR target genes, thereby modulating histone modification level and enhancing AR-induced transcriptional activity. On the other hand, PRPF6 promotes the expression of AR-FL and AR-V7 (Figure 7E). Our data demonstrated that PRPF6 plays a vital role in modulation of oncogenic AR signaling pathway and promotes the progression of prostate cancer and CRPC.

JMJD1A functions as a key coactivator for AR by epigenetic regulation of H3K9 methylation marks 31. Knockdown of PRPF6 increased histone H3K9me2 level at ARE region (Figure 4A-4C), and weakened the interaction between JMJD1A and AR (Figure 4E and 4F). Together with a stronger PRPF6-JMJD1A interaction could be detected under DHT treatment (Figure 4D), our data indicates that PRPF6 could facilitate the association of JMJD1A with AR, thereby promoting AR-mediated transcription. It is reported that PRPF6 has been known to function as a component in the protein complexes NCoR2 complex with histone deacetylase activity 27. Here, our results indicated that PRPF6, within a complex that contains JMJD1A, may contribute to AR-mediated transactivation via modulation of histone H3K9 methylation. It has also been reported that brahma related gene 1 (BRG1) associates JMJD1A as well as SET1A, a histone H3K4 methyltransferase, plays a critical role in trans-activation of *CSF1* 37. In addition to histone H3K9me2, our results showed PRPF6 knockdown resulted in a reduction in histone H3K4me3 levels at ARE region (Figure 4A-4C), implying that histone H3K4 methyltransferase may be associated with PRPF6-JMJD1A-AR complex, on the other hand, there might also be a crosstalk between demethylation of H3K9 and methylation of H3K4.

In line with the previous report that transactivation domain of PRPF6 exists within 78-495 aa residues 25, our results showed that PRPF6 full length (FL), PRPF6 5TPR (1-482 aa) and PRPF6 10TPR (1-640 aa) showed similar ability to enhance AR-FL-mediated transactivation as PRPF6 FL (Figure 3D).It is notable that truncated mutant PRPF6 15TPR (1-809 aa), which exhibited obvious speckle localization (Figure 2E), significantly enhanced AR-FL-mediated transactivation at a comparable expression level to PRPF6 FL (Figure 3D and Supplementary Figure S4). Subnuclear speckles are discovered as sites for splicing factor storage and modification 38. Increasing evidences demonstrate that, in many cases, there is a reciprocal relationship between splicing and transcription 39, 40. Studies are showing that splicing can affect chromatin organization and histone modification 41, 42. Although, relying on our data, we were unable to verify whether the increased function in transcriptional regulation of PRPF6 15TPR is related to splicing process, which remains to be elucidated in detailed future studies. Besides a cofactor of AR-FL, our data showed PRPF6 also co-activates AR-V7 mediated transcription (Figure 3), indicating that PRPF6 plays an important role in enhancing AR-FL and AR-V7 action in prostate cancer and CRPC.

AR-V7 generation could be facilitated by aberrant splicing and/or *AR* genomic structural rearrangement 3, 4, 18, 19. In addition, it has been considered that alternative splicing process of *AR-V7* couples with transcription of *AR* gene 4, 19. In line with this, levels of *AR-FL* and *AR-V7* transcripts are often tightly correlated in individual clinical specimens and xenograft models 16, 43, 44. Several splicing factors have been proposed as regulators of *AR-V7* splicing. U2AF65 and SRSF1 act as 'pioneer' factors, directing the recruitment of the spliceosome and increasing the expression of *AR-V7* 19. Histone lysine demethylase 4B (KDM4B) is phosphorylated by PKA in response to androgen deprivation, which in turn elicits its binding to U2 snRNP SF3B3, promoting *AR-V7* expression by regulating the alternative splicing of *AR* 45. PRPF6 has been demonstrated to alter the constitutive and alternative splicing of a discrete number of genes in colon cancer 24. In this study, we provided the evidence that PRPF6 also binds *cis*-regulatory elements on *AR* gene to enhancing the transcription of *AR* gene itself, thereby promoting the expression of AR-FL or AR-V7 (Figure 5). Our findings are also in good agreement with the previously proposed idea that activated steroid receptors bind to target DNA response elements and promote the recruitment of coregulators that are involved in both transcription and splicing regulation, thereby mediating effects on transcription and splice processing 46-49.

In summary, our results have demonstrated that PRPF6 regulates oncogenic AR signaling pathway in prostate cancer-derived cell lines, PRPF6 depletion reduces tumor growth of prostate cancer-derived cell lines even under the castration condition in mice, and PRPF6 is highly expressed in clinical prostate cancer samples. Our data support a previously unknown role of PRPF6 during prostate cancer progression, which may provide an insight to potential therapeutic strategies for prostate cancer, especially for CRPC.

## Supplementary Material

Supplementary figures and tables.

## Abbreviations

AR: androgen receptor
PCa: prostate cancer
AR-V7: AR-variant 7
CRPC: castration-resistant prostate cancer
ADT: androgen depletion therapy
mRNA: messenger RNA
pre-mRNA: precursor messenger RNA
snRNP: small nuclear ribonucleoprotein
PRPF6: pre-mRNA processing factor 6
CSS: charcoal/dextran-stripped serum
RNAi: RNA interference
RIP: RNA immunoprecipitation
ChIP: chromatin immunoprecipitation
IHC: immunohistochemistry
SS: splice site
DHT: dihydrotestosterone
TPR: tetratricopeptide repeat
PSA: prostate-specific antigen
KLK2: kallikrein related peptidase 2
FASN: fatty acid synthase
TMPRSS2: transmembrane protease serine 2
UBE2C: ubiquitin-conjugating enzyme E2 C
BMPRIB: bone morphogenetic protein type IB receptor
ELOVL5: ELOVL fatty acid elongase 5
HPGD: 15‐hydroxyprostaglandin dehydrogenase
SLC45A3: solute carrier family 45 a3 member
ACPP: acid phosphatase 3
ARE: AR-responsive element
SRSF: serine-arginine rich splicing factor
HNRNP: heterogeneous nuclear ribonucleoprotein
PSF/SFPQ: splicing factor proline and glutamine-rich
KDM4B: histone lysine demethylase 4B
JMJD1A: histone demethylase jumonji domain containing 1A
ASC: activating signal cointegrator
RNAPII: RNA polymerase II
