# Supplementary material for: PRPF6 promotes androgen receptor/androgen receptor-variant 7 actions in castration-resistant prostate cancer cells
Source: Int J Biol Sci. 2021 Jan 1;17(1):188–203. doi: 10.7150/ijbs.50810 (PMC7757026; doi:10.7150/ijbs.50810)
Supplement: Supplementary file 1 — Supplementary figures and tables. [file ijbsv17p0188s1.pdf]

Supplementary figure legends

Supplementary Figure S1. Validation of reduced expression of PRPF6 by lentivirus mediated knockdown.

LNCaP (left) or CWR22Rv1 (right) cells were infected with lentivirus shRNA targeting PRPF6 (shPRPF6) or control lentivirus (shCtrl). Cells were harvested for protein extraction. Western blot was performed to detect the expression of proteins using anti-PRPF6 (Santa Cruz) and GAPDH.

Supplementary Figure S2. Effects of PRPF6 knockdown on the colony formation of LNCaP cells.

LNCaP cells (10000 per well) with shPRPF6 or shCtrl were treated with 10^{-8} M DHT or ethanol for 14 days.

Supplementary Figure S3. Subcellular localizations of the PRPF6 and AR.

A and **B**. LNCaP (**A**) or CWR22Rv1 (**B**) cells were treated by 10^{-8} M DHT or ethanol vehicle for 4 hrs. Then, cells were fixed and stained with antibody against AR (Invitrogen) and PRPF6 (Proteintech). DAPI was used to visualize the nucleus (blue). Merged images were shown as indicated.

C. DU145 cells were fixed and stained with antibody against PRPF6 (Proteintech). DAPI was used to visualize the nucleus (blue). Merged images were shown as indicated.

Supplementary Figure S4 Validation of the protein expression of plasmids encoding FLAG-tagged PRPF6-FL and truncated mutants.

HEK293 cells were transfected with equal amounts of indicated plasmids or control vector. After 48 hrs, cells were collected for protein extraction and subjected to western

blot analysis using the indicated antibodies.

Supplementary Figure S5. Effect of PRPF6 knockdown on the mRNA expression of AR target genes and AR-FL in LNCaP cells.

LNCaP cells were infected with shPRPF6 or shCtrl. After treatment of 10^{-8} M DHT or ethanol vehicle for 24 hrs, cells were collected for RNA extraction and quantitative real-time PCR were performed. Student's *t*-test was performed.

Supplementary Figure S6. PRPF6 may participate in alternative splicing of *AR-V7*.

A. Diagram of *AR* gene, constitutive splicing of *AR-FL* and alternative splicing of *AR-V7*. Exons, introns, and PCR primers used to detect exon splicing are indicated.

B. Recruitments of PRPF6 on the P3b region of *AR* pre-mRNA. CWR22Rv1 cells infected with shPRPF6 or shCtrl were subjected to RIP assay with antibodies against PRPF6 (Proteintech), or normal IgG.

Supplementary Figure S7. Validation of LNCaP-AI subline.

A. Growth of parental LNCaP cells and LNCaP-AI cells under CSS condition. Cells (2500 per well) were cultured in medium with 5% CSS. After indicated time, MTS reagent was added, then absorbance at 490 nm was measured and plotted. Data were means ± SD of three independent experiments.

B. Colony formation assays of parental LNCaP cells and LNCaP-AI cells under CSS condition. The indicated numbers of cells were cultured in medium with 5% CSS for 18 days.

C. The mRNA expressions of indicated genes in parental LNCaP cells and LNCaP-AI

cells. Indicated cells were collected for RNA extraction and real-time PCR were performed.

D. The expressions of indicated proteins in parental LNCaP cells and LNCaP-AI cells. Cells were collected for protein extraction and subjected to western blot analysis using anti-AR (Proteintech). β-Actin was used as internal control.

Figure S1

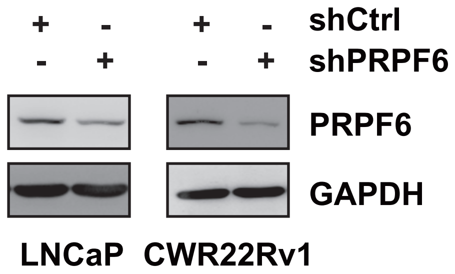

Figure S2

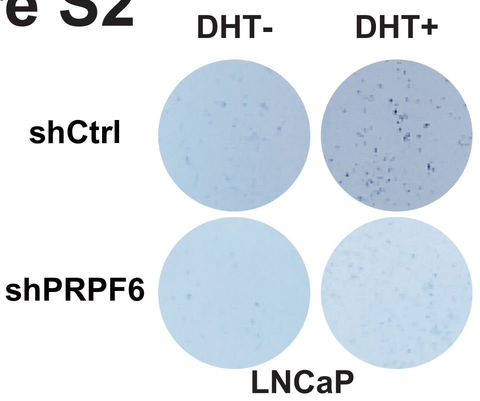

Figure S3

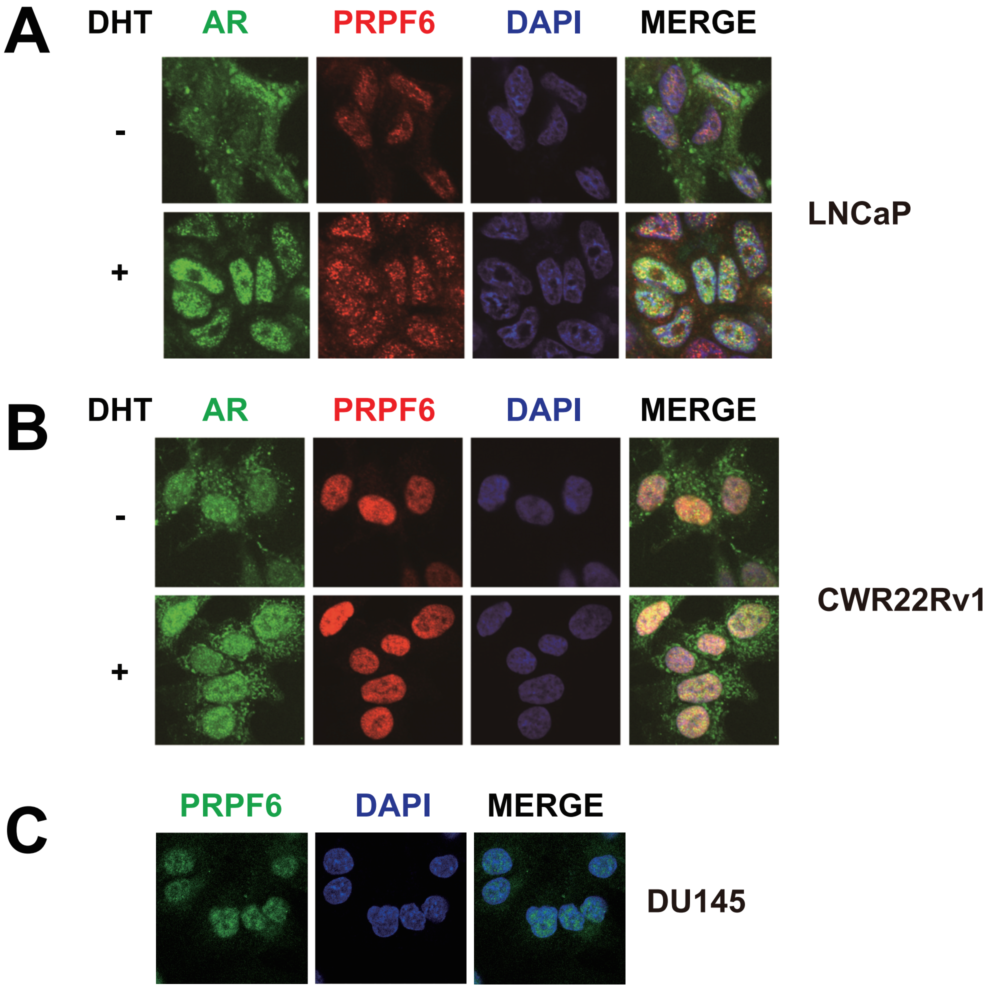

Figure S4

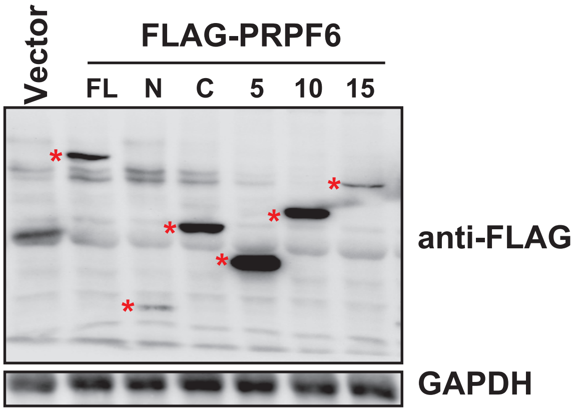

Figure S5

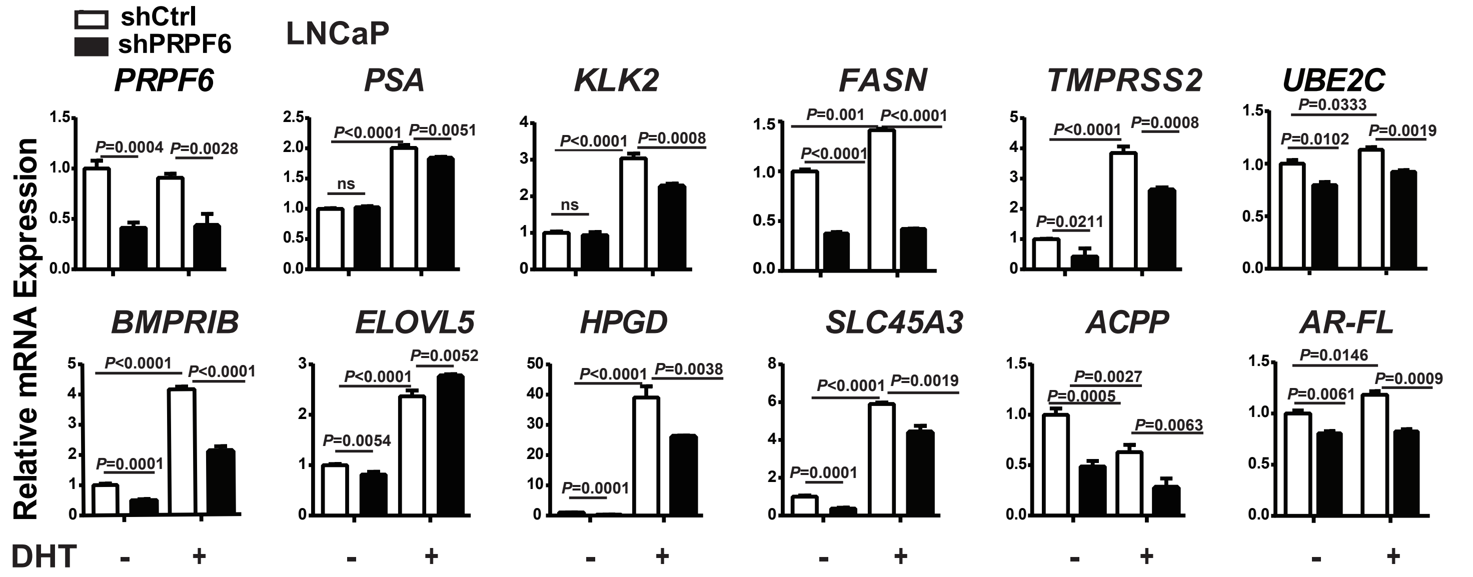

Figure S6

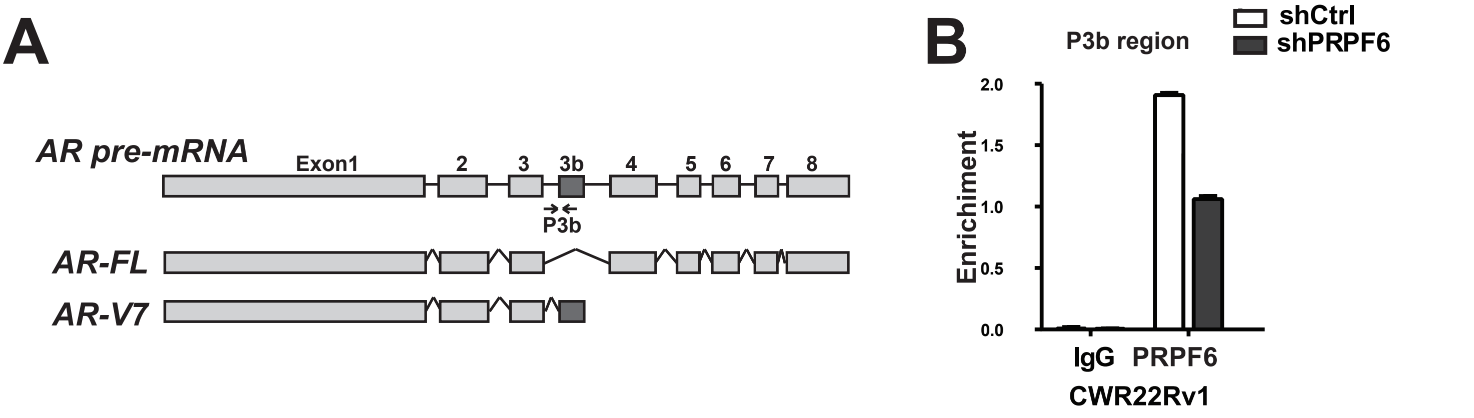

Figure S7

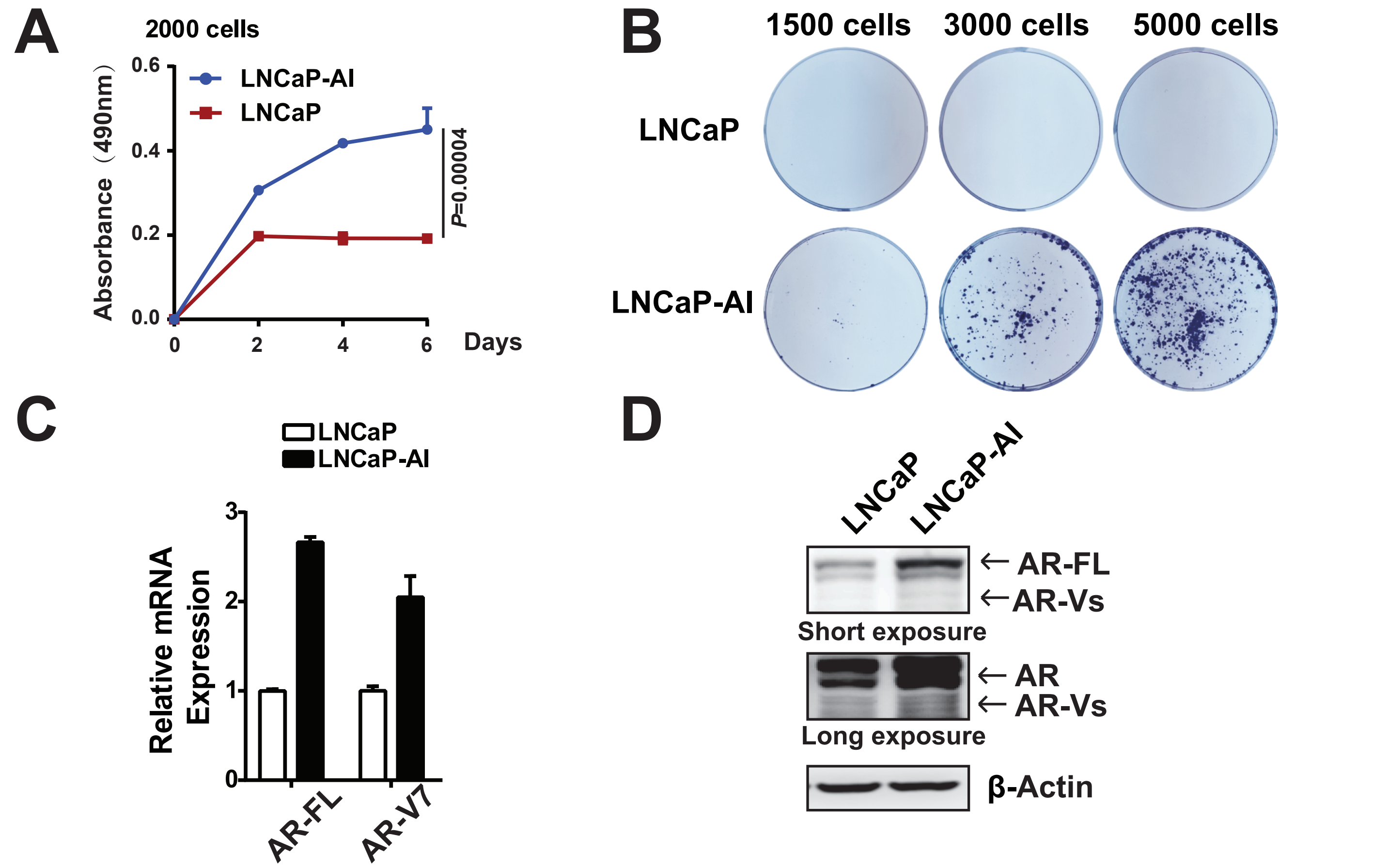

Table S1. Primers for real-time PCR

Name	Forward primer	Reverse primer
18S	5’-TTGACGGAAGGGCACCACCAG-3’	5’-GCACCACCACCCACGGAATCG-3’
PRPF6	5’-GAGGATGCTGACAGTTGTGTAG-3’	5’-CCATGGTTCTTCTCGAAGTACG-3’
AR-FL	5’-TCTTGTCGTCTTCGGAAATGT-3’	5’-AAGCCTCTCCTTCCTCCTGTA-3’
AR-V7	5’-CAGGGATGACTCTGGGAGAA-3’	5’-GCCCTCTAGAGCCCTCATTT-3’
PSA	5’-CACCTGCTCGGGTGATTCTG-3’	5’-CCACTTCCGGTAATGCACCA-3’
KLK2	5’-GCGGGTTCTGACTCTTATGCT-3’	5’-AGTGTGGGCATGAGGACTATT-3’
TMPRSS2	5’-TGAAAGCGGGTGTGAGGAGC-3’	5’-TGGTGGTGACCCTGAGTTCAA-3’
UBE2C	5’-CCATTCTGCTCTCCATCCA-3’	5’-GGCTCAACCGAGGCTTAA-3’
SLC45A3	5’-GAGCCGAGACGAAGCAGTT-3’	5’-TTAGCAGGTTGACCAGCAAGA-3’
FASN	5’-TTGTGGTCTTCTCCTCTGTGA-3’	5’-CGTTGGTGCTCATCGTCTC-3’
BMPRIB	5’-TGAGGGAGATTGTGTGCATC-3’	5’-TGAGTTTTCCCATCTGCCTT-3’
ELOVL5	5’-GTTTGTCGTCAGTCCCTTCC-3’	5’-TGGTCTGGATGATTGTCAGC-3’
HPGD	5’-AAGCAAAATGGAGGTGAAGG-3’	5’-CCAACTATGCCATGCTTTGA-3’
ACPP	5’-ACTGGTCCACGGAGTGTATG-3’	5’-CAGGTGAAGAGGTAGGAATTGC-3’

Table S2. Primers for ChIP and RIP

Name	Forward primer	Reverse primer
AREM1	5’-TAGGAATCCAACTTACAAAG GAAGTGAAGG-3’	5’-GCAGTATGGCCATTTTCACG ATATCAATTA-3’
AREM2	5’-ATATCATCCCATGGCCTCTG GCTTCCATCA-3’	5’-AGTGACACTGAGACAAATT ATAAGAAAACT-3’
PSA(AREI/II)	5’-TGGGTCTTGGAGTGCAAAGGATCT-3’	5’-AGACACGCCCAGGATGAAACAGAA-3’
PSA(AREIII)	5’-GGGGTTTGTGCCACTGGTGAG-3’	5’-GGGAGGCAATTCTCCATGGTT-3’
KLK2	5’-ACCCCTGTTGCTGTTCATCCTG-3’	5’-CCGCCCTTGCCCTGTTGG-3’
P3b	5’-ACCTCCCCAACTTTACATGCT-3’	5’-CAGGGTCTGGTCATTTTGAGA-3’
